# CRISPR/Cas9-mediated knockout of STAT1 in porcine-derived cell lines to elucidate the role of STAT1 in autophagy following classical swine fever virus infection

**DOI:** 10.3389/fimmu.2024.1468258

**Published:** 2024-10-30

**Authors:** Liyuan Zhang, Dongli Liang, Kaijun Min, Jiaxin Liang, Yu Tian, Cheng Liu, Ting Rong Luo, Xiaoning Li

**Affiliations:** ^1^ College of Animal Sciences and Veterinary Medicine, Guangxi University, Nanning, Guangxi, China; ^2^ State Key Laboratory for Conservation and Utilization of Subtropical Agro-Bioresources, Guangxi University, Nanning, Guangxi, China; ^3^ Guangxi Key Laboratory of Animal Breeding, Disease Control and Prevention, Nanning, Guangxi, China; ^4^ Guangxi Zhuang Autonomous Region Engineering Research Center of Veterinary Biologics, Guangxi University, Nanning, Guangxi, China

**Keywords:** STAT1 (Signal transducers and activators of transcription 1), CRISPR/Cas9 (Clustered regularly interspaced short palindromic repeats (CRISPR)/CRISPR-associated (Cas) systems), gene knockout cell lines, autophagy, CSFV (Classical swine fever virus), ULK1

## Abstract

Signal transducer and activator of transcription 1 (STAT1) plays a critical role in immune response, human STAT1 as a transcriptional suppressor of autophagy genes and autophagic activity. Classical swine fever virus (CSFV)-infected induce autophagy, leading to immune evasion. However, there are limited reports on the function of porcine STAT1 in autophagy during CSFV infection. There is also lack of suitable *in vitro* models for studying porcine STAT1. The objective of this study was to establish porcine PK-15 ^STAT1-/-^ and 3D4/21 ^STAT1-/-^ cell lines using the CRISPR/Cas9 system to investigate the function of the STAT1 in autophagy. The PK-15^STAT1-/-^ and 3D4/21^STAT1-/-^ cell lines, featuring homozygous knockout of STAT1 gene were successfully constructed using the CRISPR/Cas9 editing system. The knockout efficiency determined to be 82.4% and 81.1%, respectively. Infection with CSFV in porcine PK-15^STAT1-/-^ and 3D4/21^STAT1-/-^ cells led to an observable increase in autophagosomes as evidenced by transmission electron microscope. Additionally, STAT1 knockout (STAT1^-/-^) by the CRISPR/Cas9 system upregulated the expression of ULK1, Beclin1, and LC3 genes, thereby enhancing autophagy during CSFV infection. Conversely, overexpression of STAT1 downregulated the expression of ULK1, Beclin1, and LC3 genes, leading to inhibition of autophagy during CSFV infection.The application of an autophagy dual-fluorescent-tracking plasmid demonstrated that STAT1 knockout enhanced autophagy accumulation during CSFV infection, while STAT1 overexpression inhibited it. Moreover, the 3D4/21^STAT1-/-^ cell line proved to be a more suitable *in vitro* model compared to the PK-15^STAT1-/-^ cell line for elucidating the involvement of STAT1 in autophagy during CSFV infection.

## 1 Introduction

The CRISPR/Cas system is an adaptive immune mechanism in bacteria that counters invasions by identifying nucleic acids from bacteriophages or plasmids (1). This system employs the Cas9 (CRISPR associated protein 9) nuclease to cleave RNA or DNA, thereby eradicating infections (2). Known for its simplicity and rapidity, CRISPR/Cas9 has transformed the modification of endogenous genes across a wide range of species and cell types (3). A critical component of this system is the single guided RNA (sgRNA), which consists of two elements: CRISPR-derived RNA (crRNA) and trans-activating crRNA (tracrRNA) (4). Parts of the crRNA sequences are homology with tracrRNA, allowing for base pairing and formation of tracrRNA/crRNA complex. This complex precisely directs the Cas9 nuclease to the target DNA sequence for editing. Uniquely, Cas9 functions as a RNA-directed dsRNA binding protein, representing the first known factor capable of simultaneously interacting with RNA, DNA, and proteins (5).

The signal transducers and activators of transcription (STAT) family comprise seven members (STAT1, STAT2, STAT3, STAT4, STAT5a, STAT5b, and STAT6) (6). These proteins share a common structure, consisting of a DNA-binding domain, an SH2 domain, and a transcriptional activation domain at the C-terminus (7). STATs have important roles in homeostasis, development, and cell fate determination (8). STAT1 deficiency in the heart confers protection against myocardial infarction by enhancing autophagy, the conversion of LC3-I to LC3-II and the expression levels of ATG12 and Beclin1 were significantly upregulated (9). Autophagy facilitates IFN-gamma-induced JAK2-STAT1 activation and cellular inflammation (10). The activation of STAT1 resulted in IFN-γ-induced LC3B protein expression and autophagosome formation in Human immunodeficiency virus type 1 (HIV-1) infection (11). Porcine epidemic diarrhea virus (PEDV) infection upregulates the expression of TRIM28, which induces mitophagy, leading to inhibition of the JAK-STAT1 pathway (12). Given the extensive time and resources required to develop animal models lacking STAT1, and the limitations of STAT1 siRNA in fully addressing STAT1 gene transcription isoforms, constructing a STAT1-deficient cell line presents a more feasible and cost-effective approach.

Autophagy is a process in which cells degrade and recycle intracellular components (13). Unc-51-like kinase1 (ULK1), a mammalian homologue of autophagy-related gene 1 (Atg1), recruits downstream ATG complexes and directs autophagy (14). Beclin1, named Atg6, is highly conserved in eukaryotes and belong to the autophagy-related (Atg) family of proteins. Beclin1 regulates the formation of autophagy precursors and directs related proteins to locate in the autophagosome membrane (15). Microtubule-associated protein 1 light chain3 (LC3) is the first mammalian protein identified to be localized on the autophagy membrane. During autophagy, LC3 protein synthesis is clipped by Atg4 to produce cytoplasma-localized LC3-I, which is a marker protein molecule on the autophagy membrane (16). Studies have shown that HCV NS5A protein inhibits the apoptosis of cells by increasing the autophagy (17). Additionally, some reports show that the CSFV NS5A protein is linked to autophagy (18).

Classical swine fever virus (CSFV) belongs to pestivirus within the Flaviviridae family (19), which possesses a single-stranded – positive-sense RNA genome – coding for one multifunctional protein complex which is subsequently processed into: four mature structural proteins (C, E^rns^, E1, and E2) and eight non-structural proteins (N^pro^, P7, NS2, NS3, NS4A, NS4B, NS5A and NS5B) by cellular and viral proteases (20). The challenge of persistent CSFV infections poses a significant challenge to the pig breeding industry (21). Recently, studies have been carried out on the pathogenic mechanism of CSFV. For instance, studies have demonstrated that autophagy enhances the replication of CSFV *in vitro* (18). However, there are limited reports on the function of porcine STAT1 in autophagy during CSFV infection. Therefore, it is essential to construct a STAT1 knockout cell model to study the mechanism of porcine STAT1 under CSFV infection *in vitro*.

In this study, PK-15^STAT1-/-^ and 3D4/21^STAT1-/-^ cells were established using the CRISPR/Cas9 system and the results revealed that STAT1 knockout enhanced autophagyin CSFV infection, while STAT1 overexpression inhibited it.

## 2 Materials and methods

### 2.1 Cell and plasmids

The PK-15 cells were cultured in Dulbecco’s Modified Eagle’s Medium (DMEM), enriched with 10% fetal bovine serum (FBS). Concurrently, 3D4/21 cells were maintained in RPMI 1640 Medium (1×) and supplemented with 10% FBS. The cell cultures were incubated at 37°C with 5% CO_2_. The pSpCas9(BB)-2A-Puro (PX459) V2.0 vector was provided by professor TengHuang (Guangxi University, China). The vectors of porcine pEGFP-C1-LC3 and mCherry-pEGFP-LC3B were kindly donated by professor Wei Jian Huang (Guangxi University, China).

### 2.2 Main reagents

The anti-STAT1 polyclonal antibody (10144-2-AP), LC3 polyclonal antibody (14600-1-AP) and anti-Flag tag polyclonal antibodies (20543-1-AP) were purchased from Proteintech™ Co., Ltd. (Wuhan, China). Anti-CSFV E1/E2 antibody (9011) was purchased from JBT™ Co., Ltd. (Seoul, Korea), and an anti-β-actin (CW0096A) antibody was purchased from CWBIO™ Co., Ltd. (Beijing, China). The Alexa Fluor^®^488 Anti-DDDDK tag antibody (Binds to FLAG^®^ tag sequence) (ab245892) was purchased from Abcam™ Co., Ltd. (Cambridge, England). The T7E1 endonuclease (M0302S) was purchased from New England Biolabs Co., Ltd. (Beijing, China). The BbsI endonuclease (ER1011), Endotoxin free extraction Kit (A31231), and Lipofectamine 2000 (2220839) were purchased from Thermo Fisher Scientific Co., Ltd. (California, America). Puromycin (IP1280) was purchased from Solarbio Co., Ltd. (Beijing, China). DL1000 DNA Marker (3427A) was purchased from TaKaRa Co., Ltd. (Osaka, Japan). TIANamp Genomic DNA Kit (DP304-03) and 2×Taq Plus PCR MasterMix (KT205) were purchased from TIANGEN Co., Ltd. (Beijing, China). The *E.coli* strains Top10 (CD201) was purchased from Novagen Co., Ltd. (Massachusetts, USA). Immunol Fluorescence Staining Kit with Alexa Fluor 488-Labeled Goat Anti-Mouse IgG (ab150117) was purchased from Abcam Co., Ltd. (Shanghai, China). DAPI dye solution (C1005) was purchased from Beyotime Co., Ltd. (Shanghai, China). Recombinant porcine IFNα (1123-2) were purchased from Intel Phil Co., Ltd. (Anhui, China).

### 2.3 Construction of PX459 V2.0 plasmids targeting STAT1

Two different sgRNA sequences (sgRNA1: CAGCCAGCTCCCGAGCGGTT and sgRNA2: TCAGCCAGCTCCCGAGCGGT) targeted to edit the Exon 15 of porcine STAT1 gene, were separately incorporated to the PX459 V2.0 vector, and labeled PX459 V2.0-STAT1-sgRNA1 and PX459 V2.0-STAT1-sgRNA2, respectively. The specific construction steps were as follows: (1) Synthesis and annealing of oligonucleotide. Oligo1 was synthesized from sgRNA1 sequences (sgRNA1-F:

5’-*CACC* ***G*** CAGCCAGCTCCCGAGCGGTT- 3’; sgRNA1-R:

5’-*AAAC* AACCGCTCGGGAGCTGGCTG ***C***- 3’; Oligo2 was synthesized from sgRNA2 sequences (sgRNA2-F: 5’-*CACC* ***G*** TCAGCCAGCTCCCGAGCGGT- 3’; sgRNA2-R: 5’-*AAAC* ACCGCTCGGGAGCTGGCTGA ***C***- 3’) *(The line marks the BbSI cleavage site. Add “* ***C*** *” to the 3 ‘end to make the U6 promoter work effectively, with “* ***G*** *” complementing “* ***C*** *” in reverse)*, and then annealed and bonded as 1 μL of forward primer, 1 μL of reverse primer, 1 μL of T4 buffer and ddH_2_O 7 μL, heated at 37°C for 30 min, 95°C for 5 min, and naturally cooled to room temperature. (2) Plasmid linkage. The empty plasmid PX459 V2.0 was cleaved by BbSI enzyme to form a sticky end and attached to the annealed Oligo 1 or Oligo2 fragments: plasmid skeleton 3 μL, annealed Oligo1 or Oligo2 solution 9 μL, T4 ligase 1 μl, and then reacted at room temperature for 5 min. (3) Transformation of recombinant plasmid. The PCR products were then transformed in *Escherichia coli* recipient cells. Subsequently, monoclonal colonies were selected and verified by PCR and sequencing. The primer sequences used for PCR and sequencing were F:5’-TACAAAATACGTGACGTAGA-3’ and R:5’-GTTTACCGTAAATACTCCAC-3’. (4) Positive cell colonies with verified sequences were selected for expansion culture. Subsequently, PX459 V2.0-STAT1-sgRNA1 and PX459 V2.0-STAT1-sgRNA2 plasmids were isolated from these colonies using an endotoxin-free plasmid extraction kit in *Escherichia coli* recipient cells. Both plasmids were then employed for cell transfection.

### 2.4 Plasmid transfection

The PK-15 and 3D4/21 cells were cultured for 24 h before transfection, and after 80% of the cells grew in a culture plate, the cells were transfected. The PK-15 cells were transfected with PX459 V2.0-STAT1-sgRNA1 plasmid, and 3D4/21 cells were transfected with PX459 V2.0-STAT1-sgRNA2 plasmid using the LipofectamineTM 2000 Transfection Reagent following the manufacturer’s instructions, and then incubated under 37°C and 5% CO_2_ conditions.

### 2.5 Flow cytometry

The plasmid PX459 V2.0-STAT1-sgRNA1 transfected PK-15 cells and the plasmid PX459 V2.0-STAT1-sgRNA2 transfected 3D4/21 cells were digested using trypsin. The digested cells were collected and filtered for immunofluorescence flow cytometry and cell sorting. To determine transfection efficiency, cells transfected with recombinant plasmids were identified using a mouse Alexa Fluor^®^488 anti-Flag antibody. These fluorescently labeled cells were then collected by flow cytometry for analysis.

### 2.6 Indirect immunofluorescent assay

The PK-15 cells transfected with PX459 V2.0-STAT1-sgRNA1 plasmid and the 3D4/21 cells transfected with PX459 V2.0-STAT1-sgRNA2 plasmid were fixed with the methanol and acetone mixture in a 1:1 ratio for 20 min at 4°C. The fixed cells were incubated with a mouse anti-Flag antibody (1:1000) for 1 h at 37°C and then stained with a secondary antibody of goat anti-mouse IgG conjugated to Alexa Fluor 488. The images were captured using a Nikon eclipse Ti fluorescence microscope.

The PK-15 cells/PK-15^STAT1-/-^ cells or 3D4/21 cells/3D4/21^STAT1-/-^ cells were separately infected with CSFV (MOI=1). The PK-15 cells were transfected with pcDNA3.0-STAT1^-His^ vector or 3D4/21 cells were transfected with pcDNA3.0-STAT1^-His^ vector, and then infected with CSFV (MOI=1). The cells were fixed using a methanol and acetone mixture in a 1:1 ratio for 20 min at 4°C. The fixed cells were incubated with a mouse anti-CSFV E1/E2 antibody (1:300) for 1 h at 37°C and then stained with a secondary antibody of goat anti-mouse IgG conjugated to Alexa Fluor 488. The images were captured using a Nikon eclipse Ti fluorescence microscope.

### 2.7 The T7E1 enzyme digestion experiment

The recombinant plasmid transfected cells were treated with 2 μg/mL puromycin to screen the transfected positive cells, then these cells and wild-type cells were collected for DNA extraction followed by nested PCR. The amplified inner primers were STAT1-F: 5’-ATTATAATTTGAAAGTCAAA-3’, and STAT1-R:5’-CGTCAGGGCCAGCAGTGGGA -3’. The amplified outer primers were STAT1-F: 5’- CTATGAACATGACCCTATCA -3’, and STAT1-R:5’- CGTCAGGGCCAGCAGTGGGA -3’. The PCR reaction system was as follows: 1 μL of forward primer, 1 μL of reverse primer, 3 μL of DNA, 12.5 μL of 2×Taq PCR Master Mix II, 7.5 μL of ddH_2_O. The PCR reaction procedure was as follows: Pre-denaturation at 94°C for 2 min; denaturation at 98°C for 10 s, annealing at 58°C for 30 s, 34 cycles; 72°C for 5 min. Next, 0.5 μL of T7E1 enzyme was added to the reaction mixture. The mixture was then incubated at 37°C for 30 min. The cleaved products were analyzed by 2% agarose gel electrophoresis. The band intensities were quantified using ImageJ software to determine the gene knockout efficiency of the CRISPR/Cas plasmid.

### 2.8 Monoclonal cell culture and genotyping

After diluting the puromycin screened cells in the mixing pool, 200 cells were placed on each 10 cm culture plate and cultured in an incubator with 5% CO_2_ at 37°C. The cell morphology was observed every three days, and the medium was changed subsequently. Single cell clusters were labeled and single monoclonal cells were selected for culture. When monoclonal cells had grown to 80%, the cells were digested, a small number of cells were extracted for PCR for sequencing, and the remaining cells were transferred to 24-well culture plates for culture. The sequencing primers were F: 5’-ACAAAAAACAAACAAGCGTT-3’; and R: 5’-GGCAGCTCTCACTGAACCGC-3’. The PCR reaction system was as follows: 1 μL of forward primer, 1 μL of reverse primer, 3 μL of DNA, 12.5 μL of 2×Taq PCR Master Mix II, 7.5 μL of ddH_2_O. The PCR reaction procedure was as follows: pre-denaturation at 94°C for 2 min; denaturation at 98°C for 10 s, annealing at 58°C for 30 s, 34 cycles; 72°C for 5 min. The PCR products were sent to Huada BGI Company for sequencing.

### 2.9 Assessment of cellular genetic stability

Monoclonal cells were incubated at 37°C with 5% CO_2_. The total DNA was collected every 15 generations. The genetic stability was evaluated through DNA sequencing. The sequencing primers were F: 5’-ACAAAAAACAAACAAGCGTT-3’; and R: 5’-GGCAGCTCTCACTGAACCGC-3’. The PCR reaction system was as follows: 1 μL of forward primer, 1 μL of reverse primer, 3 μL of DNA, 12.5 μL of 2×Taq PCR Master Mix II, 7.5 μL of ddH_2_O. The PCR reaction procedure was as follows: pre-denaturation at 94°C for 2 min; denaturation at 98°C for 10 s, annealing at 58°C for 30 s, 34 cycles; 72°C for 5 min. The PCR products were sent to Huada BGI Company for sequencing.

### 2.10 Transmission electron microscope

The PK-15^STAT1-/-^ or 3D4/21^STAT1-/-^ cells infected with CSFV for 24 h were digested by trypsin and granulated. The cells were then suspended in a 0.1 M sodium carboxylate solution (pH 7.3) with 2% glutaraldehyde and 1% tannic acid and fixed overnight at 4°C. The cells were rinsed 3 times in sodium acetate buffer and then incubated at room temperature in 2% osmium tetroxide buffer for 2 h. The cells were then flushed with distilled water 3 times and exposed to 1% uranyl acetate water at room temperature for 15 minutes. The cells were rinsed twice in distilled water, spun into 3% agarose at 45°C and cooled into blocks. The sample was sequentially sliced with an ultra-thin slicer (Leica). The grid was stained with uranyl acetate and bismuth nitrite. Finally, prepared samples were observed and imaged with a transmission electron microscope (FEI, 80 kV).

### 2.11 Autophagy flow detection

The PK-15/PK-15^STAT1-/-^ and 3D4/21/3D4/21^STAT1-/-^ cells were initially seeded in a 12-well plate, transfected with either the pEGFP-C1-LC3 or the mCherry-pEGFP-LC3B plasmid for 6 h, and subsequently subjected to different treatments. Upon occurrence of autophagy, cells transfected with the pEGFP-C1-LC3 plasmid exhibited aggregation of green dots in the cytoplasm, indicating the presence of autophagosomes. In cases of active autophagy flux, marked by the conversion of LC3-I to LC3-II, cells transfected with the mCherry-pEGFP-LC3B plasmid exhibited the aggregation of red dots in the cytoplasm due to the fusion of autophagosomes with lysosomes to form autolysosomes. The acidic conditions within the autolysosome quench the green fluorescence while preserving the red fluorescence.

### 2.12 Transfection/infection assays

The PK-15 and 3D4/21 cells were initially seeded in the 12-well plate and then transfected with either the pcDNA3.0 plasmid or the recombinant eukaryotic expression plasmid pcDNA3.0-STAT1^-His^ encoding STAT1 gene (1.0 µg) for 6 h, and subsequently infected with CSFV (Shimen strain) at a multiplicity of infection (MOI) of 1. The supernatants and cell extracts were harvested at varying times. Cellular and viral proteins were detected by Western blot (WB) using specific antibodies. Total RNA from the infected cells was extracted using the standard TRIzol (Thermo Fisher Scientific MA, USA) RNA extraction protocol for real-time quantitative PCR (RT-qPCR).

### 2.13 WB

SDS-PAGE was conducted on a 10% acrylamide gel using the Mini-protein Tetra System (Bio-Rad, USA). The recombinant CSFV E1/E2, STAT1, and LC3 protein in acrylamide gel was visualized through Coomassie Blue R250 staining, and its molecular weight was determined using protein molecular weight markers (MBI Fermentas, Germany). Subsequently, the recombinant STAT1 protein in acrylamide gel was separated and electroblotted onto a polyvinylidene difluoride membrane (PVDF, Millipore, USA) utilizing a Semi-Dry Transfer System (Bio-Rad, USA). To minimize proteins non-specific binding, the PVDF membrane was blocked by incubating in 5% defatted milk at room temperature for 1 h. The membrane was then washed three times with TBS containing 0.05% Tween-20 (TBS-T). Primary antibodies, including polyclonal anti-STAT1 (rabbit), were applied to the membrane and incubated for 90 min at 37°C. After being washed three times with TBS-T, the membrane undergone incubation with a secondary antibody (HRP-conjugated goat anti-rabbit IgG) for 60 min at room temperature. Specific bands corresponding to the target protein were visualized using a BCIP/NBT kit purchased from Beyotime Co., Ltd. (Beijing, China).

### 2.14 RT-qPCR

The relative mRNA expressions of CSFV, STAT1, Beclin1 and ULK1were tested by RT-qPCR using specific primers and SYBR Green (**Table 1**). To measure target gene expression, total cellular RNA was isolated using the RNA simple Total RNA Kit obtained from Tiangen Biotech Co., Ltd. in Beijing, China. First-strand complementary DNA (cDNA) was synthesized using the HiScript III RT SuperMix for qPCR (+gDNA wiper) kit (Vazyme Biotech Co., Ltd., Nanjing, China) as per the manufacturer’s instructions. A LightCycler 96 PCR detection system (Roche Diagnostic Co., Ltd., Shanghai, China) was used for the quantitative assessment of CSFV, STAT1, Beclin1 and ULK1 mRNA under standard cycling conditions. GAPDH expression served as the reference gene for all reactions. Relative fold changes were calculated using the 2^−ΔΔCt^ method.

**Table 1:** Primers used in this study.

Primers	Sequence (5’-3’)	Purpose
CSFV-F	GCCATGCCCATAGTAGGACT	detection of CSFV gRNA
CSFV-R	GCTTCTGCTCACGTCGAACT	
STAT1-F	GTGTGGCAAAGAACGATCAG	detection of STAT1 mRNA
STAT1-R	TTCTGGGTAAGTTCGGTGAC	
ULK1-F	CAGACCGCCATTGACCAGAT	detection of ULK1 mRNA
ULK1-R	CCTGGGAGTGATCCCCTGAA	
Beclin1-F	CCCCTGAAACTGGACACGAG	detection of Beclin1 mRNA
Beclin1-R	GATTTTCCGCCACTATCTTCCG	
LC3-F	CAGCACCCTAGCAAGATCCC	detection of LC3 mRNA
LC3-R	GTTTCCTGGGAGGCGTAGAC	
GAPDH-F	TGGTGAAGGTCGGAGTGAAC	detection of GAPDH mRNA
GAPDH-R	GGAAGATGGTGATGGGATTTC	

### 2.15 Statistical analysis

Image J software was employed to analyze the gray levels of the electrophoretic strips and calculate the knockout efficiency. The knockout efficiency for the fragment was calculated as follows: Knockout Efficiency (%) = (Gray Level of Knocked Out Strip/(Gray Level of Knocked Out Strip + Gray Level of Unknocked Out Strip)) × 100%. SnapGene software was utilized for the analysis of sequencing peak diagrams. Data analysis was conducted using the GraphPad Prism software, version 8.0 (GraphPad Software, La Jolla, CA, USA). Differences between groups were compared using the Student’s t-test or one-way analysis of variance. All statistical analysis results were derived from comparisons between the experimental group and the Mock group. A *P* value of less than 0.05 was considered to be statistically significant.

## 3 Results

### 3.1 Design of sgRNA primers and construction of PX459 V2.0 plasmids targeting STAT1

**Figure 1:**
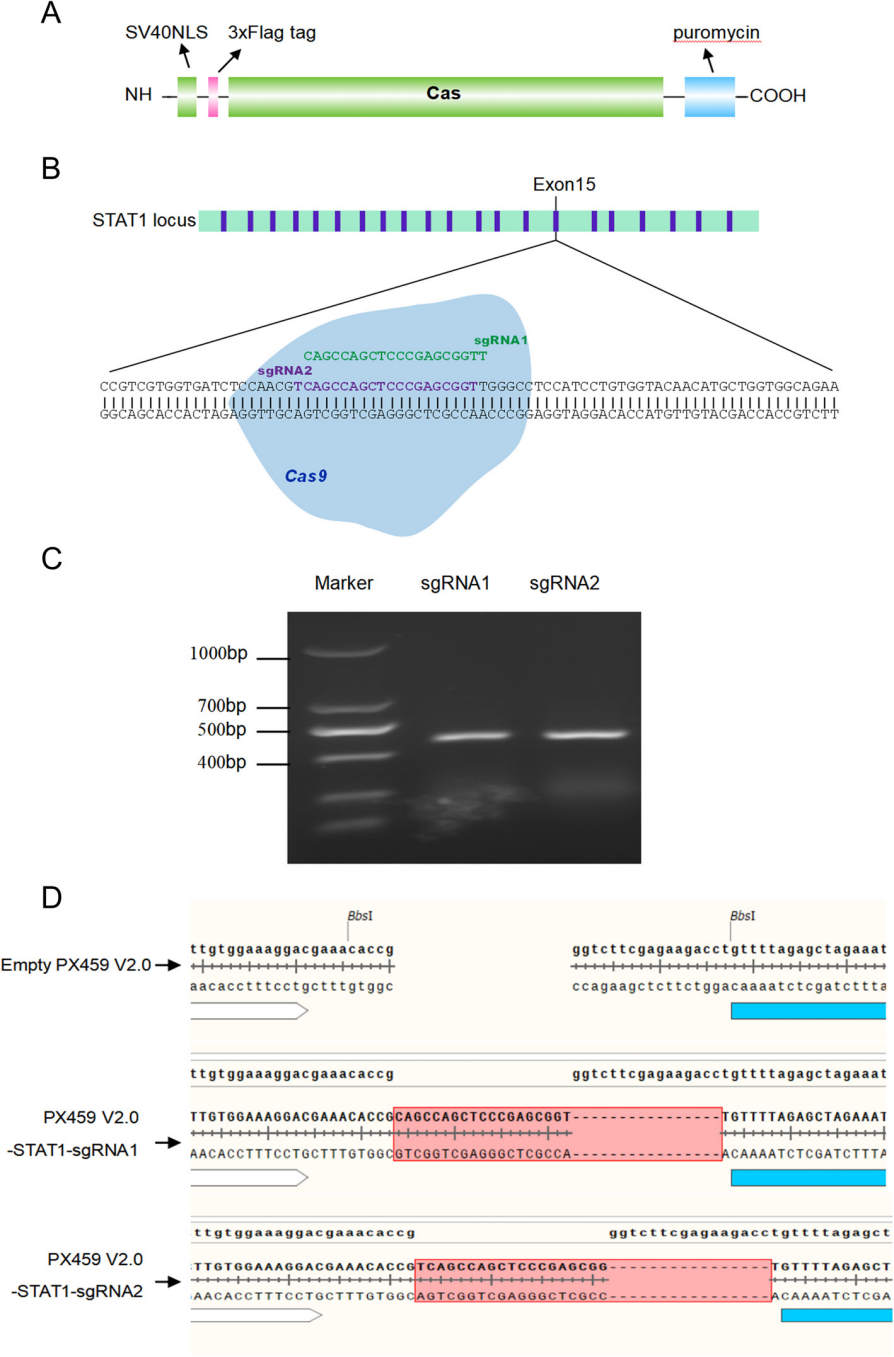
Design of sgRNA primers and construction of PX459 V2.0 plasmids targeting STAT1. **(A)** A diagram showing the Cas9 gene structure in PX459-V2.0 plasmid. The preceding segment of PX459 V2.0 vector contains a SV40 Nuclear localization sequence (SV40 NLS), a 3×Flag tag sequence, Cas9 gene and a puromycin resistance gene. **(B)** Cas9/sgRNA-mediated gene targeting of STAT1. The target sequences (the sgRNA1 in Green and sgRNA2 in Purple) contain 20 bp and PAM near the 3 ‘end, and NGG motif is required for site identification and DNA cleavage. **(C)** The annealed double-stranded sgRNA sequences were inserted into the BbSI-treated PX459 V2.0 linear vectors, named PX459-V2.0- STAT1-sgRNA1 and PX459-V2.0-STAT1-sgRNA2, respectively. The sgRNA target sequences from PX459-V2.0-STAT1-sgRNA1 and PX459-V2.0-STAT1-sgRNA2 plasmids were amplified by PCR. **(D)** Recombinant vectors PX459-V2.0-STAT1-sgRNA1 and PX459-V2.0-STAT1-sgRNA2 transformed *Escherichia coli* cells top10, and then the plasmid was extracted for further sequencing.

CRISPR-Cas9 is a revolutionary gene editing tool that uses engineered molecules like scissors to precisely alter a specific section of an organism’s DNA. This allows scientists to perform gene knockout, insert new genetic information, or making precise changes to a gene’s sequence (4). The preceding segment of PX459 V2.0 vector contains a SV40 Nuclear localization sequence (SV40 NLS), a Triple-Flag (3×Flag) tag sequence, Cas9 gene, and a puromycin resistance gene (**Figure 1A**). Cas9 can recognize target sequences containing PAM (Protospacer adjacent motif, NGG motif) under the action of single guide RNA (sgRNA), and realize double strand break of DNA sequence. Two pairs of sgRNA sequences (named sgRNA1 and sgRNA2) targeting exon 15 of STAT1 gene were selected as shown in **Figure 1B**. Two pairs of sgRNA sequence primers design website refer to CRISPOR http://crispor.tefor.net/ and sgRNA sequence primers shown in “2.3” (named Oligo1 and Oligo2). CRISPR/Cas9 system PX459 V2.0 vector was cleaved using BbSI enzyme to form sticky ends, and then separately connected with sgRNA1 and sgRNA2 sequences to form PX459 V2.0-STAT1-sgRNA1, and PX459 V2.0-STAT1-sgRNA2, respectively. The specific primers used are detailed in section ‘2.3’ and are visualized in **Figure 1C**. Following amplification, the PCR products were analyzed on an agarose gel, revealing a specific band at 465 bp. This band corresponds to the DNA fragment containing the sgRNA sequence. In addition, sequencing results shown in **Figure 1D** confirmed that both pairs of sgRNA sequences were successfully inserted into the PX459 V2.0 vector. These results demonstrated that the PX459 V2.0-STAT1-sgRNA1 and PX459 V2.0-STAT1-sgRNA2 plasmids targeting STAT1 were successfully constructed.

### 3.2 Recombinant plasmid transfection efficiency in PK-15 cells and 3D4/21 cells

**Figure 2:**
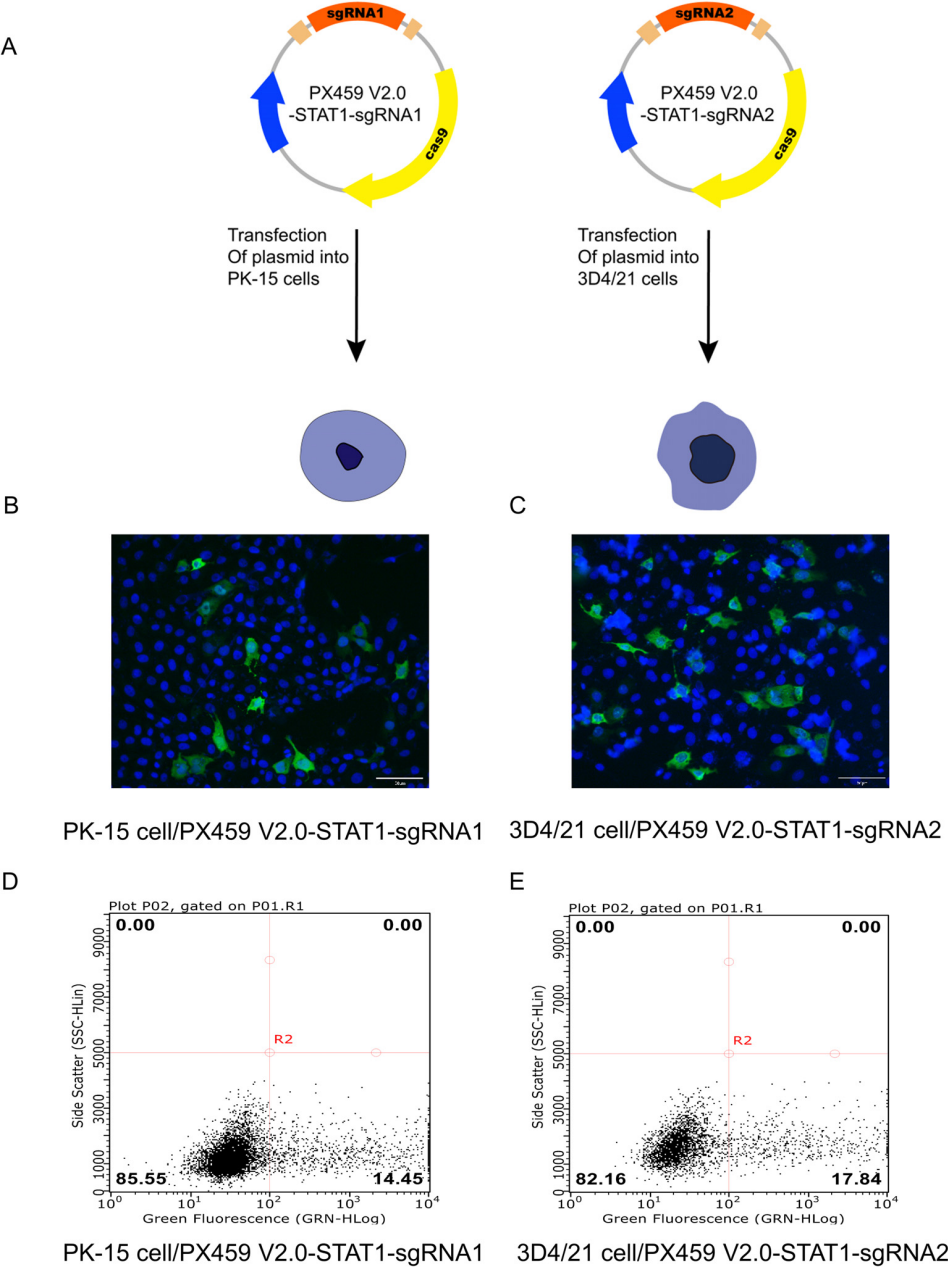
Detection of recombinant plasmid transfection efficiency of porcine PK-15 cells and 3D4/21 cells. **(A)** The transfection strategies of PX459 V2.0-STAT1-sgRNA1 and PX459 V2.0-STAT1-sgRNA2 plasmids. **(B, C)** Green fluorescence was detected in PX459 V2.0-STAT1-sgRNA1 transfected PK-15 cells **(B)** and PX459 V2.0-STAT1-sgRNA2 transfected 3D4/21 cells **(C)** using an anti-Flag antibody at 24 hpi by IFA to evaluate the transfection efficiency. **(D, E)** Green fluorescence was detected in PX459 V2.0-STAT1-sgRNA1 transfected PK-15 cells **(D)** and PX459 V2.0-STAT1-sgRNA12 transfected 3D4/21 cells **(E)** using Alexa Fluor^®^488 Anti-DDDDK tag antibody by Flow cytometry (FCM) to assess the transfection efficiency.

Our above experiments confirmed that the recombinant plasmids PX459 V2.0-STAT1-sgRNA1 and PX459 V2.0-STAT1-sgRNA2 containing designed sgRNA1 and sgRNA2 targeting STAT1 were successfully constructed. The transfection efficiency of recombinant plasmids expressing Triple-Flag tagged STAT1 protein was determined using two methods: immunofluorescence assay (IFA) and flow cytometry. PX459 V2.0-STAT1-sgRNA1 plasmid was transfected into PK-15 cells, while PX459 V2.0-STAT1-sgRNA2 plasmids was used for 3D4/21 cells. **Figure 2A** showed the transfection strategies of PX459-STAT1-sgRNA1 and PX459-STAT1-sgRNA2 plasmids. Results of IFA revealed green fluorescence in PK-15 and 3D4/21 cells transfected with either PX459 V2.0-STAT1-sgRNA1 (**Figure 2B**) or PX459 V2.0-STAT1-sgRNA2 plasmids (**Figure 2C**). A mouse anti-Flag antibody was used as the primary antibody. These observations indicate that transfection successfully led to the expression of sgRNA sequences targeting the STAT1 gene fused with a triple-Flag tag in both PK-15 and 3D4/21 cells. Additionally, cells successfully transfected with recombinant plasmids were detected by flow cytometry and cell sorting using a mouse Alexa Fluor^®^488 Anti-Flag antibody as primary antibody. The results demonstrated that the successfully transfected cells accounted for 14.45% of the total number of PK-15 cells (**Figure 2D**) and 17.84% of the total number of 3D4/21 cells (**Figure 2E**), respectively. These results indicated that both PX459-STAT1-sgRNA1 and PX459-STAT1-sgRNA2 plasmids could be stably expressed in porcine PK-15 cells and 3D4/21 cells.

### 3.3 STAT1 knockout efficiency in PK-15^STAT1-/-^ and 3D4/21^STAT1-/-^ polyclonal cells

**Figure 3:**
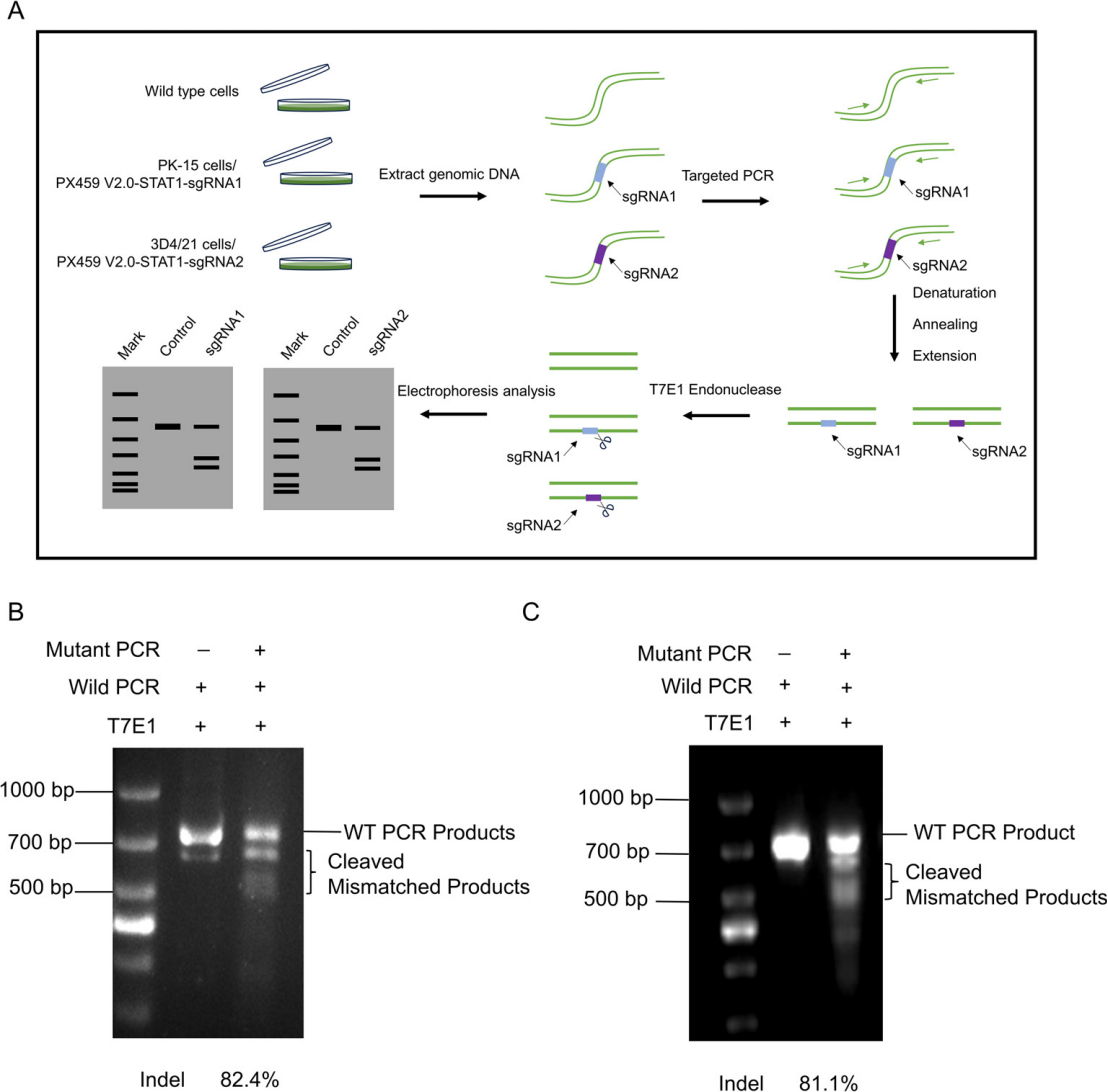
Detection of knockout efficiency of PK-15^STAT1-/-^ and 3D4/21^STAT1-/-^ polyclonal cells. **(A)** The procedure of T7E1 enzyme digestion. (1) The genomes extracted from wild-type and transfected cell lines. (2) PCR amplified DNA fragments containing the mutation site, and the mutation site should not be located in the center of the PCR fragment, to ensure that the two bands of different sizes would be cut. (3) Hybridization of wild type DNA with mutant DNA PCR products was performed. (4) T7E1 enzyme was added to the hybrid system and reacted at 37°C for 30 min. (5) Analysis of enzyme digestion by 2% agarose gel electrophoresis. **(B)** The knockout efficiency of PK-15^STAT1-/-^ cells were analyzed by 2% agarose gel electrophoresis and the gray value was calculated by the ImageJ software. **(C)** Analysis of the knockout efficiency of 3D4/21^STAT1-/-^ cells by 2% agarose gel electrophoresis and the gray value was measured by ImageJ software.

Given that PX459-STAT1-sgRNA1 and PX459-STAT1-sgRNA2 plasmids can be stably expressed in porcine PK-15 cells and 3D4/21 cells, to further determine the editing efficiency of STAT1 gene by the two pairs of sgRNA, we transfected PX459 V2.0-STAT1-sgRNA1 plasmid was into PK-15 cells to obtain PK-15^STAT1-/-^ polyclonal cells, and PX459 V2.0-STAT1-sgRNA2 plasmid was transfected into 3D4/21 cells to obtain 3D4/21^STAT1-/-^ polyclonal cells. DNA was extracted from both polyclonal cells and wild-type cells, with the latter serving as a negative control. Finally, the wild-type cell DNA was mixed with the polyclonal cell DNA (also known as mutated cell DNA) and reacted with the T7E1 enzyme. As shown in **Figure 3A**, cell DNA was collected from wild-type, the PK-15, and 3D4/21 cells separately and transfected with PX459 V2.0-STAT1-sgRNA1 and PX459 V2.0-STAT1-sgRNA2 plasmids for 24 h. PCR was used to amplify the sequence near the target site, wild type PCR products were hybridized with mutant PCR products according to the “2.7” system. Then, T7E1 enzyme was added to the PCR product and the reaction was done at 37°C for 30 min. As shown in **Figure 3B**, PCR results showed the presence of specific bands at 720 bp in wild-type cell DNA. However, the hybrid DNA of wild-type and mutant cells displayed specific bands at 720 bp as well as two additional fragments cleaved by BbSI endonuclease. Gray value statistics showed that knockout efficiency of the STAT1 gene fragment in PK-15^STAT1-/-^polyclonal cells was 82.4%. Similarly, as displayed in **Figure 3C**, PCR results showed the presence of specific bands at 720 bp in wild-type cell DNA. However, hybrid DNA from wild-type and mutant showed specific bands at 720 bp, along with two additional BbSI restriction fragments. Gray-value statistics showed that knockout efficiency of the STAT1 gene fragment was 81.1% in 3D4/21^STAT1-/-^polyclonal cells. The formula of Knockdown Efficiency was as per the “2.13”.

The results showed that the Cas9 enzyme precisely edited the target gene STAT1, leading to reverse complementation of the wild-type template and mutant template, which was cut by the mismatch base recognized by T7E1 enzyme, to induce the frame-shift mutation of STAT1 gene. The knockout efficiencies of PK-15^STAT1-/-^ and 3D4/21^STAT1-/-^ polyclonal cells have been confirmed to be very high.

### 3.4 The monoclonal cells PK-15^STAT1-/-^ and 3D4/21^STAT1-/-^ were screened and sequenced

**Figure 4:**
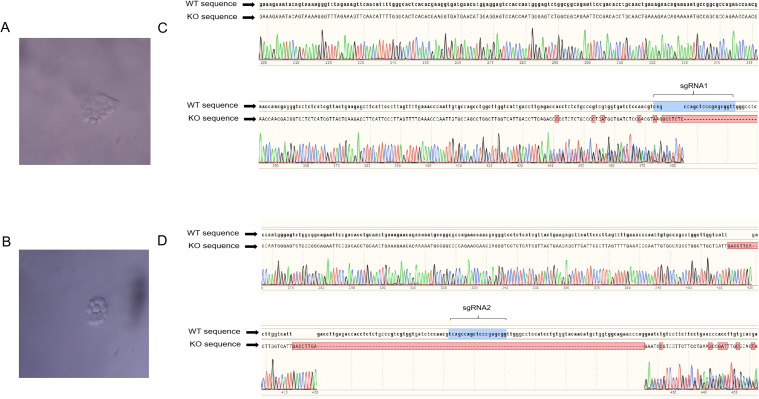
The monoclonal cells PK-15^STAT1-/-^ and 3D4/21^STAT1-/-^ were screened, cultured and sequenced. **(A)** PK-15^STAT1-/-^ monoclonal cell culture. **(B)** The DNA of PK-15^STAT1-/-^ monoclonal cell lines extracted for further sequencing. **(C)** 3D4/21^STAT1-/-^ monoclonal cell culture. **(D)** The sequencing analysis of DNA of 3D4/21^STAT1-/-^ monoclonal cell lines.

These results indicated that the sgRNA sequences targeting STAT1 gene were effective in PK-15^STAT1-/-^ and 3D4/21^STAT1-/-^ polyclonal cells. To obtain stable expression cell lines of PK-15 ^STAT1-/-^ and 3D4/21 ^STAT1-/-^ cells, the polyclonal cells were screened using 2 μg/mL of puromycin. After diluting the puromycin-screened cells on culture plate, the monoclonal cells were selected for expanded culture. As shown in **Figures 4A, B**, PK-15 ^STAT1-/-^ and 3D4/21 ^STAT1-/-^ monoclonal cells were selected and cultured on 96-well plates, respectively. Once the number of monoclonal cells increased to 1 × 10^6^, some monoclonal cells were subjected to DNA extraction, and the sequence was amplified by PCR near the target site for sequencing verification, the PCR system and procedure were identified as per “2.8”. The remaining cells were transferred to cell bottles for further culture. The sequencing results identified overlapping peaks near the sgRNA target region and subsequent sequences in PK-15 ^STAT1-/-^ cells (**Figure 4C**) and 3D4/21 ^STAT1-/-^ cells (**Figure 4D**), indicating PK-15 ^STAT1-/-^ and 3D4/21 ^STAT1-/-^ monoclonal cell lines were successfully acquired.

### 3.5 The mRNA and protein expression of STAT1 in PK-15^STAT1-/-^ and 3D4/21^STAT1-/-^ cells

**Figure 5:**
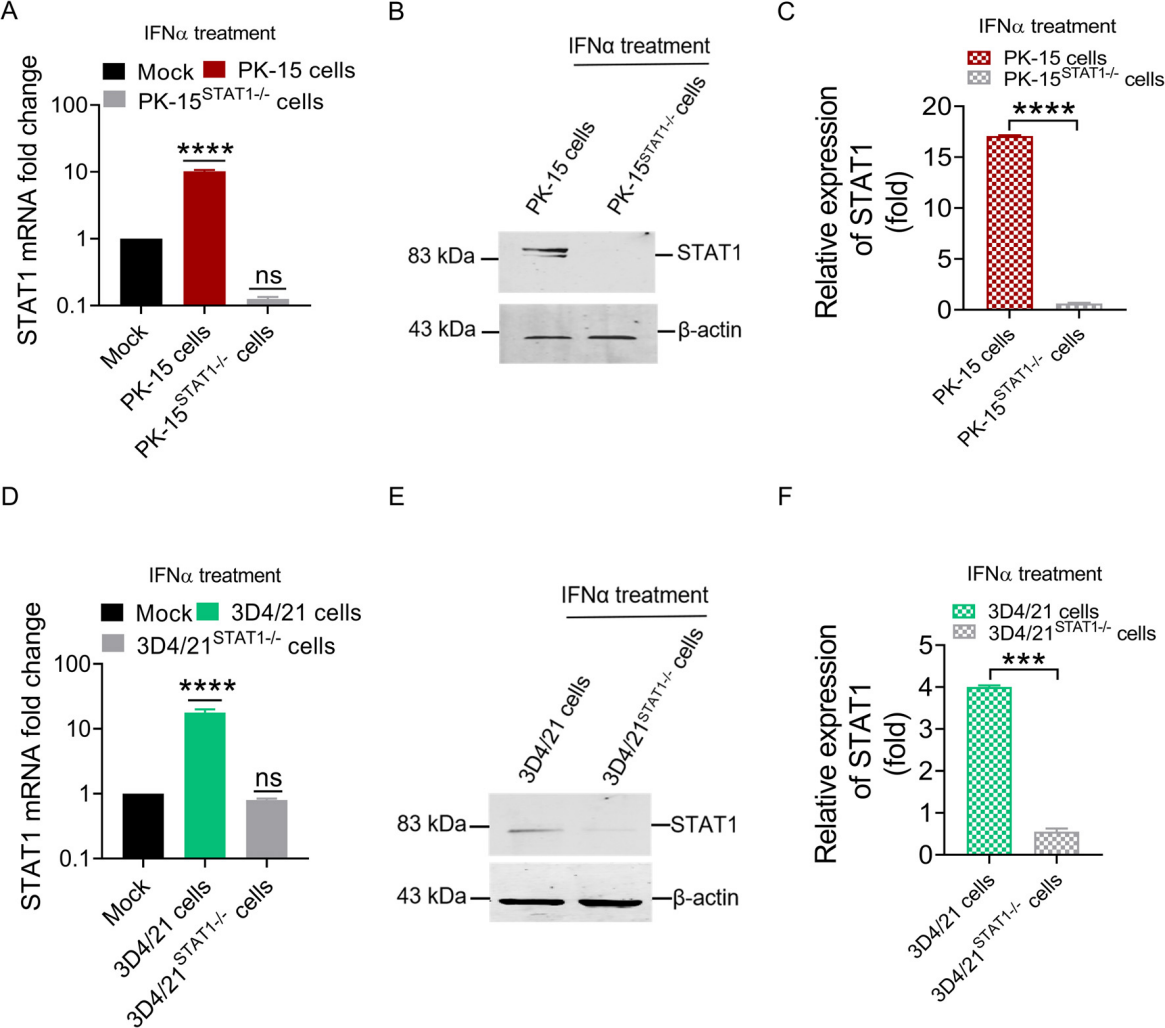
The mRNA and protein expression of STAT1 could not be detected in PK-15^STAT1-/-^ cells and 3D4/21^STAT1-/-^ cells. **(A, B)** PK-15 wild-type cells and PK-15^STAT1-/-^ cells were stimulated with 100 IU/mL IFNα, respectively, and cell lysates of the treated cells were collected at 48 h for RT-qPCR **(A)** and Western blot analysis **(B)**. **(D, E)** 3D4/21 wild-type cells and 3D4/21^STAT1-/-^ cells were stimulated with 100 IU/mL IFNα, respectively, and the cell lysates of the treated cells were collected at 48 h for RT-qPCR **(D)** and Western blot analysis **(E)**. **(C, F)** The gray values of STAT1 band from WB were measured by comparing them with β-actin using ImageJ software (no significance, ns; *** *P* < 0.001, **** *P* < 0.0001).

After screening, culture, and sequencing of PK-15^STAT1-/-^ and 3D4/21^STAT1-/-^ cells, the mRNA and protein expression levels of STAT1 were measured in wild-type cell, PK-15 ^STAT1^ **^-/-^** cell and 3D4/21 ^STAT1^ **^-/-^** cell treated with a final concentration of 100 IU/ml IFNα by RT-qPCR and Western blot. As shown in **Figure 5A**, treatment with 100 IU/ml IFNα upregulated STAT1 mRNA expression up to 10.1 folds (*P*<0.0001) in wild-type PK-15 cells at 48 h. However, the mRNA levels of STAT1 were not detected in PK-15 ^STAT1^ **^-/-^** cells treated with IFNα at 48 h. Simultaneously, the expression of STAT1 protein was detected in wild-type PK-15 cells treated with IFNα by Western blot. However, the STAT1 protein was not detected in the PK-15 ^STAT1^ **^-/-^** cells treated with IFNα at 48 h (**Figures 5B, C**).

In 3D4/21 ^STAT1^ **^-/-^** cells treated with 100 IU/ml IFNα, STAT1 mRNA level was significantly increased in wild-type 3D4/21 cells, by up to 10.3 folds (*P*<0.0001) at 48 h, but undetectable in 3D4/21 ^STAT1^ **^-/-^** cells treated with IFNα (**Figure 5D**). Similarly, Western blot analysis revealed STAT1 protein expression in wild-type 3D4/21 cells treated with IFNα, but due to the characteristic of macrophages polarization, only very weak band could be detected in 3D4/21^STAT1-/-^ cells (**Figure 5E**). Gray value analysis of STAT1 bands in 3D4/21^STAT1-/-^ cells was performed. The results showed that STAT1 protein decreased significantly in 3D4/21^STAT1-/-^ cells treated with IFNα at 48 h compared to the wild-type group (*P*<0.001) (**Figure 5F**). These results confirm successful knockout of the STAT1 gene in both PK-15 and 3D4/21 cells.

### 3.6 STAT1 knockout enhanced autophagy during CSFV infection

To explore the effect of STAT1 on autophagy in CSFV infection, PK-15 ^STAT1^ **^-/-^** cell and 3D4/21 ^STAT1^ **^-/-^** cell were infected with 1 MOI CSFV. The autophagosome were observed by TEM, as well as the protein levels of CSFV E2, STAT1, LC3-I, and LC3-II were detected by WB.

**Figure 6:**
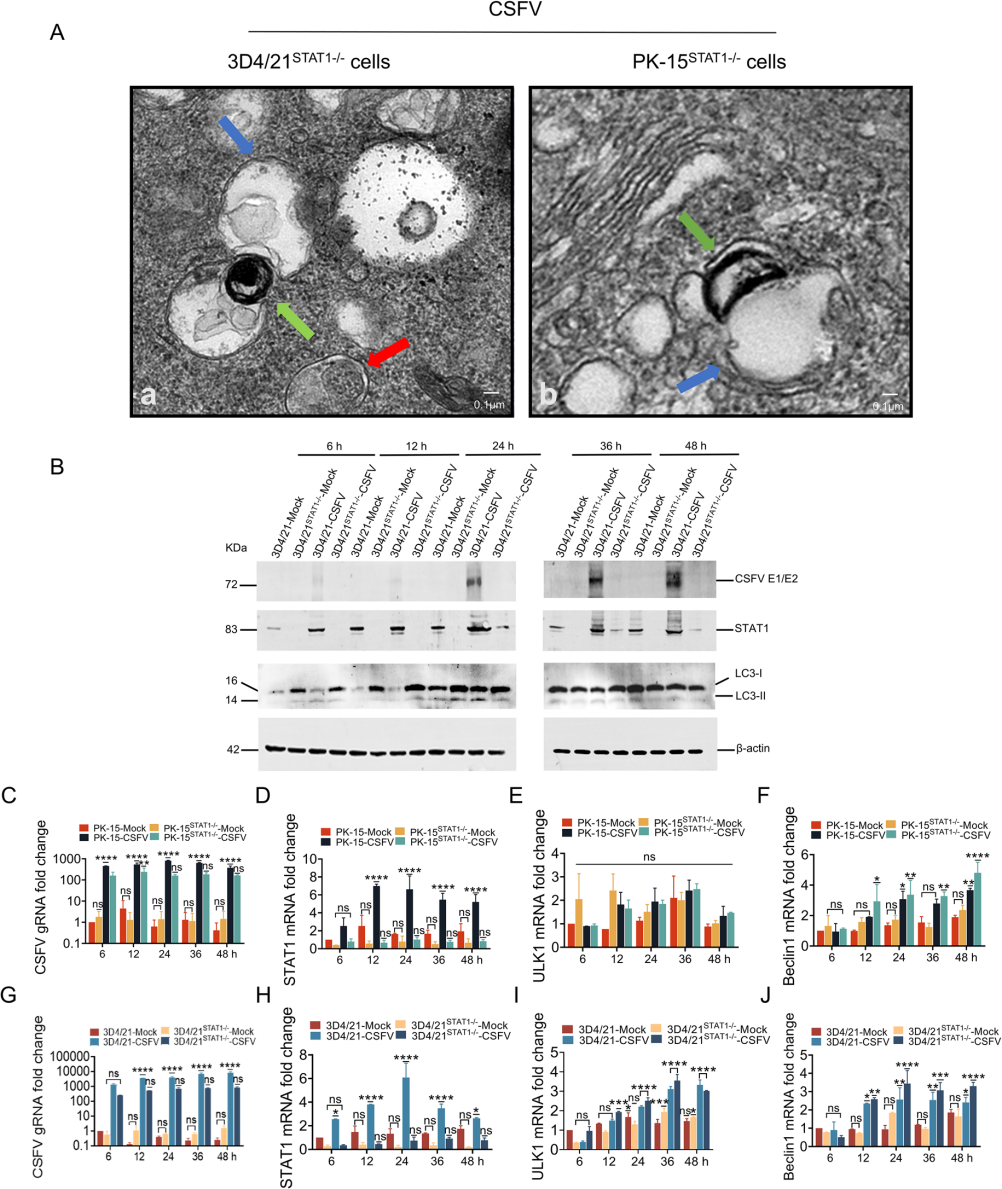
STAT1 gene knockout (STAT1-/-) enhanced autophagy during CSFV infection. **(A)** PK-15^STAT1-/-^ cells or 3D4/21^STAT1-/-^ cells were infected with 1 MOI CSFV, then cultured for 24 h. The cells were then processed for TEM as described under Materials and Methods 2.9. The blue arrows indicated the monolayer autophagosomes; the green arrows indicated the lysosome; the red arrow indicated bilayer autophagosomes. **(B)** The protein levels of CSFV E1/E2, STAT1, and LC3 were evaluated in 3D4/21 cells or 3D4/21^STAT1-/-^ cells separately infected with the CSFV Shimen strain (MOI = 1) at 6, 12, 24, 36, and 48 h by WB analysis. **(C–F)** The gRNA level of CSFV and mRNA levels of STAT1, ULK1 and Beclin1 were assessed in PK-15 cells or PK-15^STAT1-/-^ cells separately infected with the CSFV Shimen strain (MOI = 1) at 6, 12, 24, 36, and 48 h by RT-qPCR analysis. **(G-J)** The gRNA level of CSFV and mRNA levels of STAT1, ULK1 and Beclin1 were assessed in 3D4/21 cells or 3D4/21^STAT1-/-^ cells separately infected with the CSFV Shimen strain (MOI = 1) at 6, 12, 24, 36, and 48 h by RT-qPCR analysis. (no significance, ns; *P<0.05, **P<0.01, ***P<0.001, ****P<0.0001).

Autophagy induction was initially investigated by TEM. The results showed that in STAT1-knockout cells (PK-15 ^STAT1^ **^-/-^** cell or 3D4/21 ^STAT1^ **^-/-^** cell) infected with 1 MOI CSFV, there was an accumulation of monolayer autophagosomes (blue arrows) and lysosomes (green arrows) during fusion, as well as the observation of bilayer autophagosomes (red arrows) that did not fuse with lysosomes, was also noted (**Figure 6A**). Subsequently, we also determined the levels of autophagic markers LC3 protein. 3D4/21 cells knockout for STAT1 showed increased LC3 levels compared to normal 3D4/21 cells, with LC3-I transformation to LC3-II protein observed at 24 hours post infection (hpi) (**Figure 6B**). However, LC3 protein expression was not detected in PK-15 cells or PK-15^STAT1-/-^ cells.

The above results indicated that the knockout of STAT1 enhanced CSFV infection-induced autophagy. The current study addresses the role of human STAT1 as a transcriptional suppressor of autophagy genes and autophagic activity (22). To further investigate the impact of STAT1-knockout on the autophagy gene transcriptional levels during CSFV infection, PK-15 ^STAT1-/-^ cells and 3D4/21 ^STAT1-/-^ cells were infected with 1 MOI of CSFV. The genomic RNA (gRNA) level of CSFV and mRNA levels of STAT1, ULK1, and Beclin1 were assessed using RT-qPCR. In PK-15 cells infected with 1 MOI CSFV, the mRNA levels of STAT1, ULK1 and Beclin1 increased with the viral replication (**Figures 6C–F**). In PK-15 ^STAT1^ **^-/-^** cells infected with 1 MOI CSFV, the STAT1 mRNA expression was significantly suppressed. Specifically, the STAT1 mRNA expression decreased by 3.4-, 11.5-, 6.5-, 7.7-, and 6.3-fold compared to PK-15 cells infected with 1 MOI CSFV (control group) at 6, 12, 24, 36, and 48 hpi, respectively (**Figure 6D**). A significant decrease in the levels of CSFV gRNA was observed to be 2.8-, 2.2-, 5.0-, 3.4-, and 2.3-fold at 6, 12, 24, 36, and 48 hpi, respectively, compared to control group (**Figure 6C**). Moreover, compared to control group, it was observed that the mRNA level of ULK1 was slight increased at 36 and 48 hpi (**Figure 6E**); Beclin1 mRNA level was increased by 1.1-, 1.1-, and 1.3-fold at 24, 36, and 48 hpi, respectively (**Figure 6F**).

In 3D4/21 cells infected with 1 MOI CSFV, STAT1, ULK1 and Beclin1 mRNA levels increased with viral replication (**Figures 6G–J**). In 3D4/21^STAT1^ **^-/-^** cells infected with 1 MOI CSFV, STAT1 mRNA expression was exhibited significant suppression. Specifically, the STAT1 mRNA expression decreased by 8.3-, 9.2-, 8.5-, 4.2-, and 3.7-fold compared with 3D4/21 cells infected with 1 MOI CSFV (control group) at 6, 12, 24, 36, and 48 hpi, respectively; while STAT1 protein levels could not be detected in 3D4/21^STAT1^ **^-/-^** cells infected with CSFV (**Figures 6B, H**). Compared with the control group, a significant decrease in CSFV gRNA levels was observed at 6-, 12-, 24-, 36-, and 48- hpi, with fold reductions of 5.4, 7.0, 5.7, 9.5, and 10.2, respectively (**Figure 6G**). CSFV E2 protein was undetectable (**Figure 6B**). Furthermore, ULK1 mRNA levels were increased by 3.0-, 1.2-, 1.1-, and 1.1-fold at 6, 12, 24, and 36 hpi compared with the control group (**Figure 6I**). Beclin1 mRNA levels showed a similar increase at 24, 36, and 48 hpi (1.3-, 1.2-, and 1.3-fold increase, respectively) (**Figure 6J**).

These findings suggested that the knockout of STAT1 enhances autophagy during CSFV infection.

### 3.7 STAT1 gene knockout (STAT1-/-) enhanced autophagy flow during CSFV infection

To further investigate the impact of STAT1 knockout on autophagy flow, the pEGFP-C1-LC3 and mCherry-pEGFP-LC3B vectors were transfected into 3D4/21^STAT1-/-^ and PK-15^STAT1-/-^ cells, respectively. The dynamic changes of autophagy flow in living cells were observed using fluorescence microscopy.

**Figure 7:**
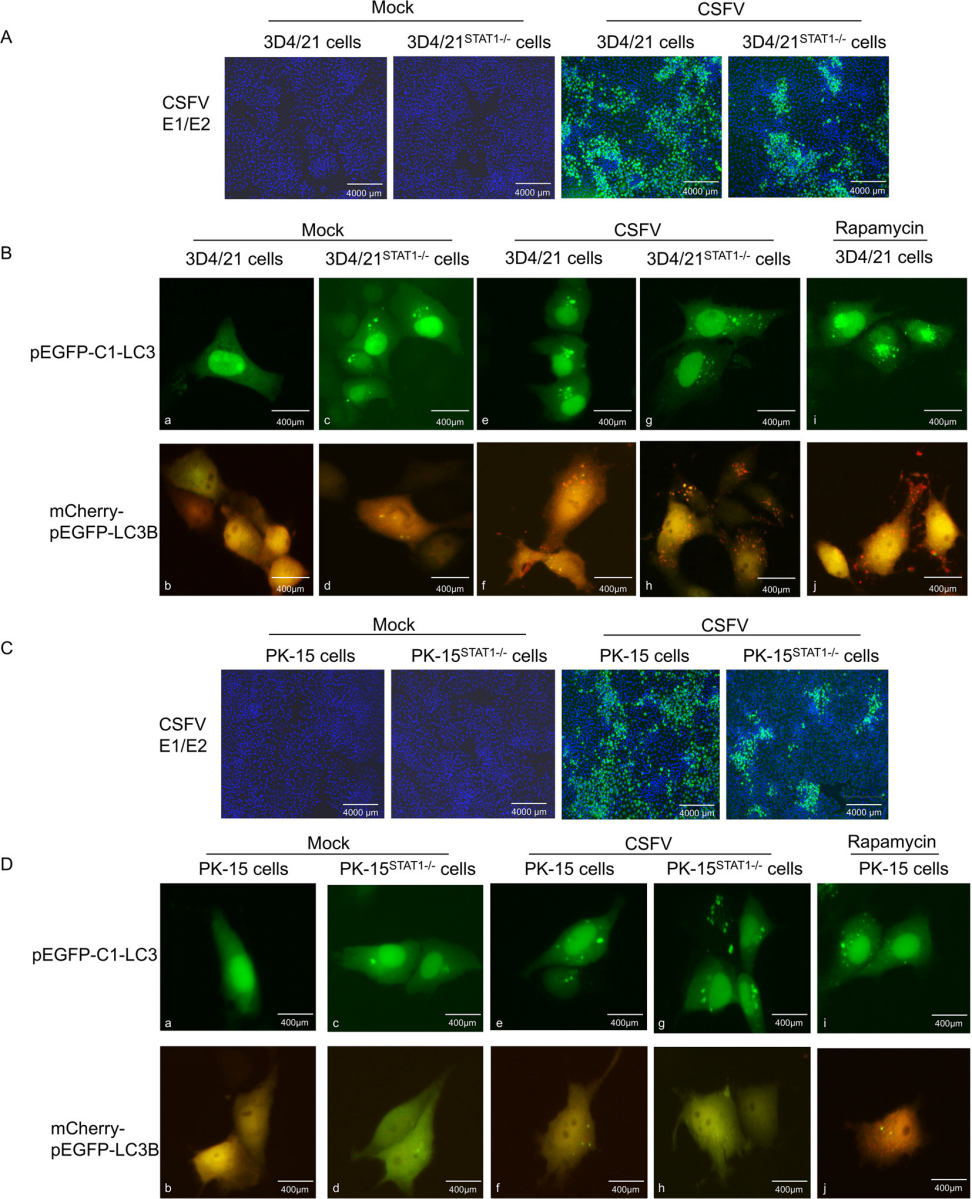
STAT1 gene knockout (STAT1-/-) enhanced autophagy flow during CSFV infection. The pEGFP-C1-LC3 and mCherry-pEGFP-LC3B plasmids were separately transfected into 3D4/21 cells or 3D4/21^STAT1-/-^ cells for 6 h, and then infected with CSFV (MOI=1). **(A)** CSFV replication in 3D4/21 cells and 3D4/21^STAT1-/-^ cells. **(B)** Autophagy flow was examined using fluorescence microscope at 24 (h) The pEGFP-C1-LC3 and mCherry-pEGFP-LC3B plasmids were separately transfected into PK-15 cells or PK-15^STAT1-/-^ cells for 6 h, and subsequently infected with CSFV (MOI=1). **(C)** CSFV replication in PK-15 cells and PK-15^STAT1-/-^ cells. **(D)** Autophagy flow was observed using a fluorescence microscope at 24 h. (no significance, ns; * *P* < 0.05, **** *P* < 0.01, *** *P* < 0.001, **** *P* < 0.0001).

Initially, the vectors of pEGFP-C1-LC3 and mCherry-pEGFP-LC3B were transfected into 3D4/21^STAT1-/-^ cells. In the Mock groups, STAT1 knockout maintained basal autophagy in the absence of external stimulation compared with normal cells, thereby upregulating LC3-I expression (**Figure 7B**a, c). However, these autophagosomes did not mature into complete autophagic structures. Instead, only yellow fluorescent puncta were observed in the cytoplasm. This suggests that STAT1 knockout triggered the formation of a limited number of autophagosomes, but these autophagosomes failed to fuse with lysosomes and complete the autophagy process (as described in section 2.12) (**Figure 7B**b, d). In the CSFV infection groups, compared with mock, CSFV replicated in both normal 3D4/21 cells and STAT1-knockout 3D4/21 cells (**Figure 7A**). Additionally, the results showed that the virus induced autophagy in 3D4/21 and 3D4/21^STAT1-/-^ cells, leading to LC3-I accumulation within the cytoplasm. However, CSFV upregulated the expression level of LC3-I in 3D4/21^STAT1-/-^ cells than in 3D4/21cells (**Figure 7B**e, g). Moreover, the complete autophagy induced by CSFV-infected 3D4/21^STAT1-/-^ cells significantly exceeded that of the CSFV-infected 3D4/21 cells, probably due to the higher number of red fluorescent spots in the cytoplasm (**Figure 7B**f, h). Similarly, when stimulated with Rapamycin as a positive control, LC3-I accumulation and complete autophagy detected in the cytoplasm (**Figure 7B**i, j).

Next, the vectors of pEGFP-C1-LC3 and mCherry-pEGFP-LC3B were transfected into PK-15^STAT1-/-^ cells. The changes in autophagy flow in living cells were examined through fluorescence microscopy. In the Mock groups, STAT1 knocking out led to basal autophagy levels in PK-15 cells compared to normal cells. This resulted in increased LC3-I expression (**Figure 7D**a, c) without inducing complete autophagy (**Figure 7D**b, d). Moreover, the PK-15^STAT1-/-^ cells exhibited much lower overall autophagy activity compared to 3D4/21^STAT1-/-^ cells. In the CSFV infection groups, compared with mock, CSFV replicated in both normal PK-15 cells and STAT1-knockout PK-15 cells (**Figure 7C**). Interestingly, both normal PK-15 cells and PK-15^STAT1-/-^ cells displayed some level of autophagy upon CSFV infection, as evidenced by increased LC3-I accumulation in the CSFV-infected group (**Figure 7D**e, g). However, it was difficult to detect autophagy flow in the cytoplasm of normal PK-15 cells and PK-15^STAT1-/-^ cells; only yellow fluorescent spots of autophagy were observed (**Figure 7D**f, h). Even under Rapamycin stimulation, only a few red fluorescent spots of complete autophagy were observed in cytoplasm (**Figure 7D**i, j).

These results suggested that STAT1 knockout enhanced autophagy flux under CSFV infection. In comparison to PK-15 cells, 3D4/21 cells serve as a superior model for studying autophagy due to their classification as immune cells. These cells are actively involved in both non-specific and specific immune responses, exhibiting heightened sensitivity to pathogen invasion. Furthermore, autophagy is more likely to occur in immune cells, serving as a crucial mechanism for host cells to eliminate pathogens.

### 3.8 Overexpression of STAT1 inhibited the expression of autophagy genes

The above results indicated that STAT1 knockout enhanced autophagy following CSFV infection. To further explore the effect of STAT1 on key autophagy-related genes, the pcDNA3.0-STAT1^-His^ eukaryotic vector was separately transfected into CSFV-infected PK-15 cells and CSFV-infected 3D4/21 cells. Subsequently, the gRNA level of CSFV and mRNA levels of STAT1, ULK1, Beclin1, and LC3 were quantified by RT-qPCR, and the protein levels of CSFV E2, STAT1, LC3-I, and LC3-II were measured by WB.

**Figure 8:**
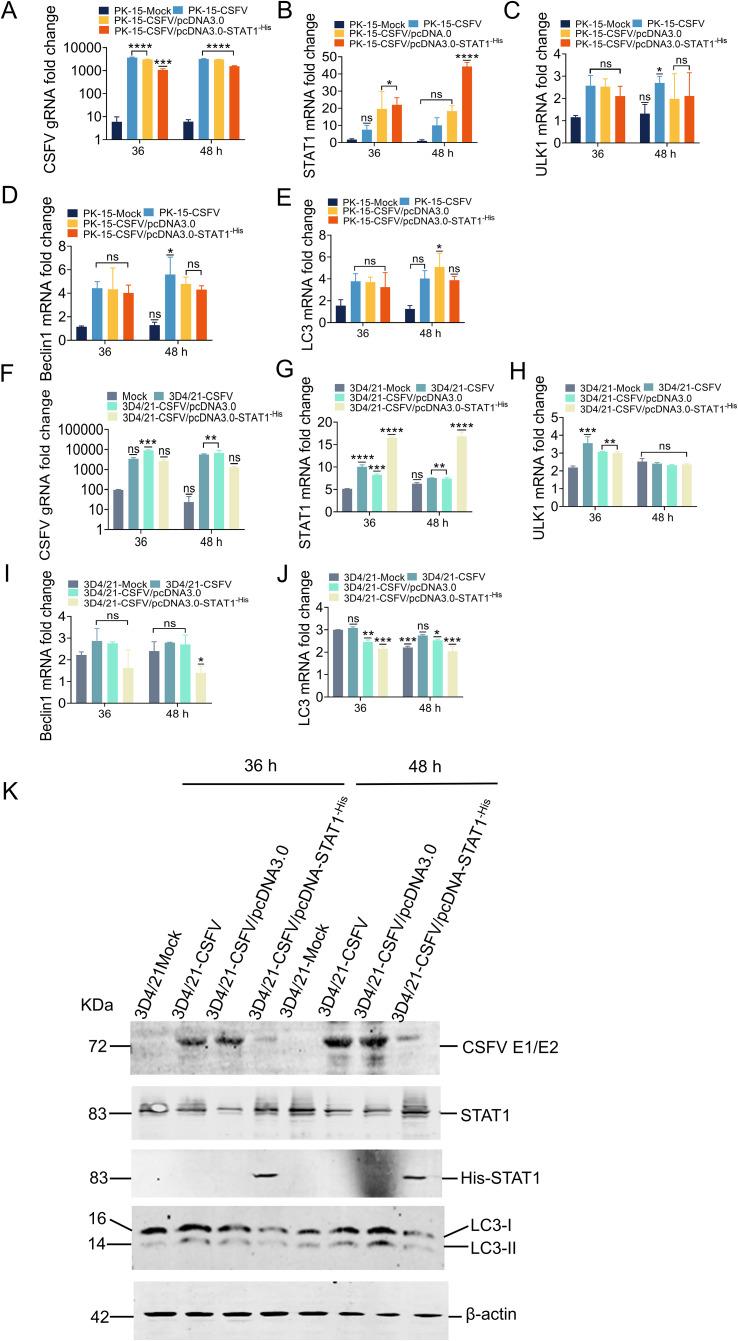
Overexpression of STAT1 inhibited the expression of autophagy genes. **(A-E)** The gRNA level of CSFV and mRNA levels of STAT1, ULK1, Beclin1, and LC3 were measured by RT-qPCR analysis at 36 and 48 h in PK-15 cells transfected with the pcDNA3.0-STAT1^-His^ vector then infected with the CSFV Shimen strain (MOI=1). **(F-J)** The gRNA level of CSFV and mRNA levels of STAT1, ULK1, Beclin1, and LC3 were quantified by RT-qPCR analysis at 36 and 48 h in 3D4/21 cells transfected with the pcDNA3.0-STAT1^-His^ vector and infected with the CSFV Shimen strain (MOI=1). (no significance, ns; *P<0.05, **P<0.01, ***P<0.001, ****P<0.0001). **(K)** The protein levels of CSFV E1/E2, STAT1, and LC3 were evaluated by WB analysis in 3D4/21 cells transfected with the pcDNA3.0-STAT1^-His^ vector then infected with the CSFV Shimen strain (MOI=1).

In PK-15 cells transfected with 1.0 μg empty vector and then infected with 1 MOI CSFV (empty vector group), STAT1, ULK1, Beclin1, and LC3 expression increased with viral replication (**Figures 8A–E**). In PK-15 cells transfected with 1.0 μg pcDNA3.0-STAT1^-His^ vector and then with CSFV (MOI=1), This caused a significant increase in the exogenous mRNA expression of STAT1. Specifically, the STAT1 mRNA expression was increased by 1.9- and 2.0-fold compared to empty vector group at 36 and 48 h, respectively (**Figure 8B**). On the contrary, CSFV gRNA decreased by 3.0- and 1.9-fold at 36 and 48 h compared with the empty vector group (**Figure 8A**). Similarly, compared with the empty vector group, ULK1 mRNA levels were decreased by 1.2-fold at 36 h (**Figure 8C**). The mRNA levels of Beclin1 were slight decreased, while LC3 mRNA levels were decreased by 1.1- and 1.3-fold at 36 and 48 h (**Figures 8D, E**).

In 3D4/21 cells transfected with 1.0 μg empty vector and infected with 1 MOI CSFV (empty vector group), the expression of STAT1, ULK1, Beclin1, and LC3 increased with viral replication (**Figures 8F–J**). In 3D4/21 cells transfected with 1.0 μg pcDNA3.0-STAT1^-His^ vector and infected with CSFV (MOI=1), the exogenous mRNA expression of STAT1 gene was significantly enhanced. Specifically, the STAT1 mRNA expression was increased by 2.0- and 2.3-fold compared to empty vector group at 36 and 48 h, respectively; while STAT1 protein levels were significantly increased (**Figures 8G, K**). Our analysis revealed a significant decrease in both CSFV gRNA and protein levels following CSFV infection. Specifically, compared to the empty vector group, CSFV gRNA levels were reduced by 3.4- and 5.3-fold at 36 and 48 h, respectively (**Figure 8F**). CSFV E2 protein levels were also downregulated at both 36 and 48 h (**Figure 8K**). Similarly, compared to empty vector group, ULK1 mRNA levels were slight decreased (**Figure 8H**); Beclin1 mRNA levels were decreased by 1.7- and 2.0-fold (**Figure 8I**). Finally, LC3 mRNA levels were downregulated by 1.1- and 1.3-fold at 36 and 48 h, respectively. LC3 protein levels also showed a significant decrease compared to the empty vector group. In detail, the expression of LC3-I was significantly reduced following addition of exogenous STAT1, and no transformation of LC3-I to LC3-II was observed at 36 and 48 h (**Figures 8J, K**). These results suggested that STAT1 is involved in autophagy during CSFV infection.

### 3.9 Overexpression of STAT1 inhibited the autophagy flow during CSFV infection

To further investigate the role of STAT1 overexpression on autophagy flow, the pEGFP-C1-LC3/pcDNA3.0-STAT1^-His^ and mCherry-pEGFP-LC3B/pcDNA3.0-STAT1^-His^ vectors were transfected into 3D4/21 and PK-15 cells, respectively. The changes of autophagy flow in living cells were examined by fluorescence microscopy.

**Figure 9:**
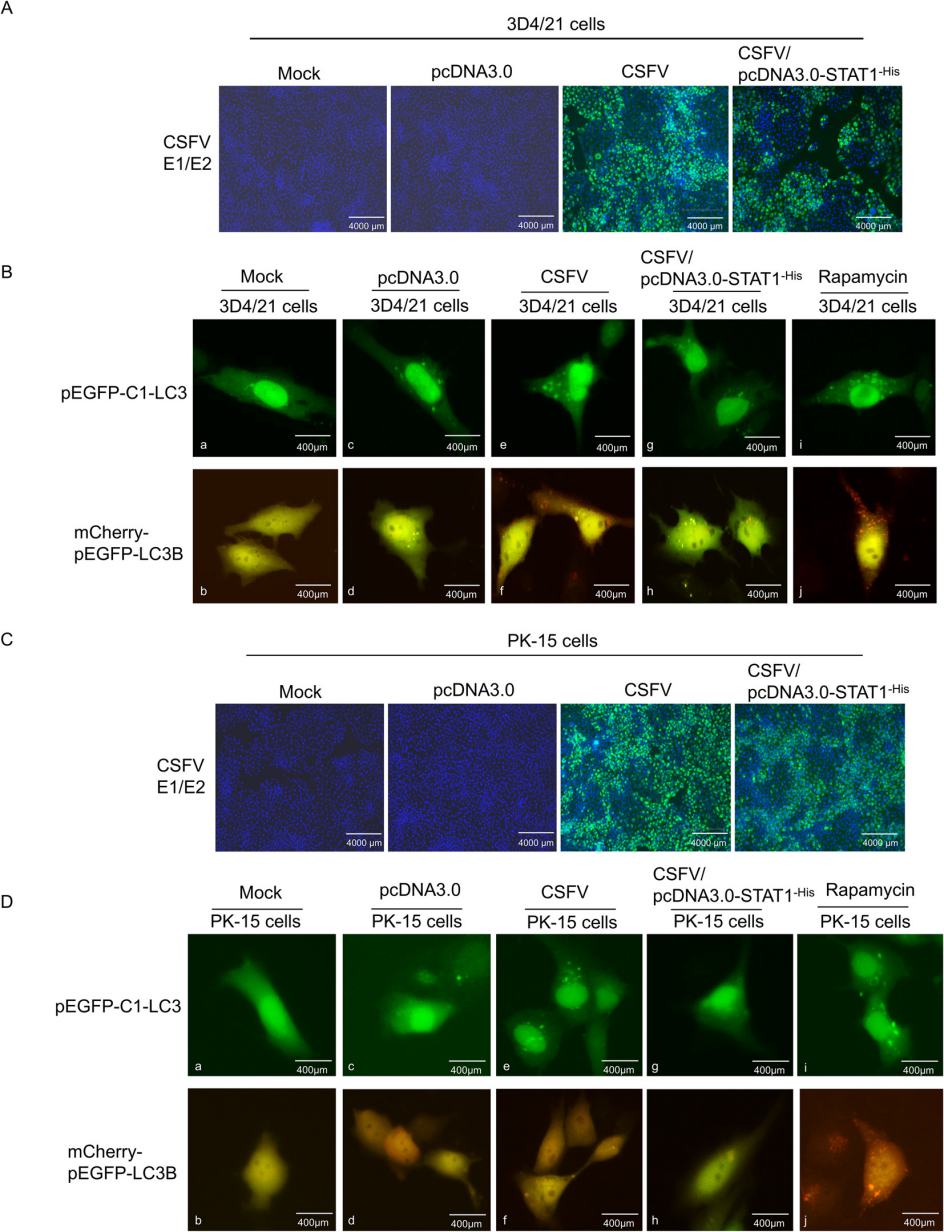
Overexpression of STAT1 inhibited autophagy flow under CSFV infection. The pEGFP-C1-LC3/pcDNA3.0-STAT1^-His^ and mCherry-pEGFP-LC3B/pcDNA3.0-STAT1^-His^ plasmids were separately transfected into 3D4/21 cells for 6 h, and subsequently infected with CSFV (MOI=1). **(A)** CSFV replication in 3D4/21 cells and 3D4/21 cells transfected with pcDNA3.0-STAT1^-His^ vector. **(B)** Autophagy flow was using a fluorescent microscope at 24 **(h)** The pEGFP-C1-LC3/pcDNA3.0-STAT1^-His^ and mCherry-EGFP-LC3B/pcDNA3.0-STAT1^-His^ plasmids were separately transfected into PK-15 cells for 6 h, and subsequently infected with CSFV (MOI=1). **(C)** CSFV replication in PK-15 cells and PK-15 cells transfected with pcDNA3.0-STAT1^-His^ vector. **(D)** Autophagy flow was observed under fluorescence microscopy at 24 h.

Initially, the pEGFP-C1-LC3/pcDNA3.0-STAT1^-His^ and mCherry-pEGFP-LC3B/pcDNA3.0-STAT1^-His^ vectors were separately transfected into 3D4/21 cells. Transfection with empty plasmid resulted in up-regulated levels of LC3-I compared to the Mock group (**Figure 9B**a, c). Empty vector transfection resulted in the formation of a limited number of autophagosomes, which appeared as yellow fluorescent puncta in the cytoplasm. However, these autophagosomes did not mature into complete autophagic structures. This suggests that empty vector transfection triggered autophagosome formation, but these autophagosomes failed to fuse with lysosomes, a critical step for complete autophagy (**Figure 9B**b, d). In the CSFV infection groups, compared to the mock and empty vector groups, CSFV was found to replicate in both 3D4/21 cells and 3D4/21 cells transfected with the pcDNA3.0-STAT1^-His^ vector (**Figure 9A**). Overexpression of STAT1 significantly inhibited the upregulation of LC3-I induced by CSFV infection compared to the CSFV-infected group. This inhibition resulted in a marked reduction in the fusion of autophagosomes with lysosomes. Consequently, only a few red fluorescent puncta were observed in the cytoplasm (**Figure 9B**e-h). In contrast, stimulated with Rapamycin as a positive control, LC3-I accumulation and complete autophagy induction (red autophagy fluorescence spots) were also observed in the cytoplasm (**Figure 9B**i, j).

Subsequently, the pEGFP-C1-LC3/pcDNA3.0-STATA^-His^ and mCherry-pEGFP-LC3B/pcDNA3.0-STATA^-His^ vectors were separately transfected into PK-15 cells. Neither LC3-I nor LC3-II puncta were observed in either the Mock group or the empty plasmid transfection group (**Figure 9D**a-d). This suggests that basal autophagy levels in PK-15 cells were significantly lower than those in 3D4/21 cells. In the CSFV infection groups, compared to the mock and empty vector groups, CSFV was found to replicate in both PK-15 cells and PK-15 cells transfected with the pcDNA3.0-STAT1^-His^ vector (**Figure 9C**). Similar to the results in 3D4/21 cells, overexpression of STAT1 also inhibited the up-regulation of LC3-I induced by CSFV infection in PK-l5 cells, significantly reducing complete autophagy (**Figure 9D**e-h). In contrast, stimulated with Rapamycin as a positive control, LC3-I accumulation and complete autophagy induction were also observed in the cytoplasm (**Figure 9D**i, j).

These findings showed that STAT1 overexpression reduced autophagy flow induced by CSFV infection, and 3D4/21 cells serve as a better model for studying autophagy than PK-l5 cells.

## 4 Discussion

Originally developed as an immune mechanism for bacteria and archaea to defend against viral invasion, the CRISPR/Cas9 system has evolved into a simple and fast gene editing tool that can efficiently modify endogenous genes across a wide variety of species and cell types (23). In 2016, Whitworth et al. edited exon 7 of porcine CD163 gene through CRISPR/Cas technology, reducing the risk of porcine reproductive and respiratory syndrome virus (PRRSV) infection in pigs (24). In 2017, Niu et al. knocked out the endogenous retrovirus (PERV) sequence in the porcine gene through CRISPR/Cas9 technology and cloned several PERV inactivated piglets, which made a big step towards the use of pig organs in human organ transfer (25). In 2020, Li et al. utilized the CRISPR/Cas9 system to integrate a large transgenic cassette (20 kbp) carrying various digestive enzyme genes into the cep112 site of porcine fetal fibroblasts (PFFs). This approach established an improved model for the expression of exogenous digestive enzymes in porcine saliva (26).

The STAT1 performs the following functions, acting as an anti-infection, inhibiting cell proliferation, regulating the immune system, enabling cell differentiation, inhibiting tumor growth, inhibiting cell growth, and promoting cell apoptosis (27, 28). Mutation and knockdown of STAT1 gene help in the study of the infection and disease pathogenesis. Loss of STAT1 protects hair cells from ototoxicity through modulation of STAT3, c-Jun, Akt, and autophagy factors (29). STAT1 contributes to the loss of irreplaceable cardiac myocytes by increasing apoptosis and reducing cardioprotective autophagy (30). Currently, most studies focus on STAT1 gene use inhibitors to explore its role in humans and mice, with some focusing on reporting on the function of porcine STAT1. In addition, current methods of inhibitor, silencing, knockdown, and interference employing existing technologies are not specific, incomplete or unable to silence STAT1 gene expression, hence its critical to develop a pig STAT1-knockout cell line that can achieve complete silencing and long-term stability *in vitro* culture, laying a foundation for further research on the role of STAT1 in the pathogenesis of virus infection.

Autophagy is a powerful tool that host cells use to defend against viral infection. Double-membrane vesicles, and termed autophagosomes deliver trapped viral cargo to the lysosome for degradation. Autophagy initiates an innate immune response by cooperating with pattern recognition receptor signaling to induce interferon production (31). Research has shown that CSFV infection inhibits the phosphorylation of MTOR (mechanistic target of rapamycin kinase), thereby leading to autophagy initiation (32). CSFV restricts necroptosis to sustain infection by inducing autophagy/mitophagy-targeted degradation of RIPK3 (33).

Although CSFV can proliferate in various cell types, except pig kidney cells, no cell type has shown high sensitivity to virus proliferation (34). The PK-15 cell line, originating from porcine kidneys, is instrumental in understanding CSFV proliferation and characteristics, playing a critical role in veterinary vaccine research and production (35). The 3D4/21 cell line, derived from porcine alveolar macrophages, is commonly utilized in studies concerning immunity and infection. Macrophages play diverse roles in the immune system depending on their polarization states. For instance, they regulate the production of pro-inflammatory factors such as IFNγ, TNFα, and IL-6 when polarize into M1 macrophages, and regulate the production of anti-inflammatory factors such as IL-10, IL-4, and TGFβ1) when polarize into M2 macrophages (36). While substantial advancements have been made in the functional study of STAT1, its specific interaction with CSFV warrants further investigation.

Therefore, in this study, the CRISPR/Cas9 editing system was used to knock out the STAT1 gene in PK-15 cells and 3D4/21 cells, to establish good cell models for investigating the CSFV infection. The PX459 V2.0-STAT1-sgRNA1 and PX459 V2.0-STAT1-sgRNA2 plasmids targeting exon 15 of the STAT1 gene were transfected into PK-15 cells and 3D4/21 cells, respectively, and the polyclone cells were further enriched through puromycin screening. RT-qPCR and Western blot analyses revealed no STAT1 mRNA or protein expression in the knockout cells with homozygous fragment deletion. This confirmed that STAT1 knockout successfully eliminated its expression at both the mRNA and protein levels in the generated cells. In addition, loss of STAT1 resulted in upregulation of LC3 and autophagy flow, while STAT1 overexpression inhibited autophagy and autophagy flow. Recent studies have demonstrated that human STAT1 could bind to a putative regulatory sequence in the 5’-flanking of ULK1, and the deficiency of STAT1 alters this sequence, thereby increasing the activity of the ULK1 promoter (22). Similarly, STAT1 negatively regulated transcriptional levels of ULK1 and Beclin1 genes in CSFV infection, and when STAT1 increased, the mRNA levels of ULK1 and Beclin1 genes were inhibited; while STAT1 decreased, the mRNA levels of ULK1 and Beclin1 genes were increased.

STAT1 plays a crucial role in maintaining the balance between apoptosis and autophagy (37). CSFV infection upregulates STAT1 and induce autophagy (**Figure 6**). Despite the established role of autophagy in CSFV replication, the specific function of STAT1 in the regulation of autophagy during this viral infection remains largely unexplored. Similarly, our findings demonstrated that STAT1 knockout increased autophagy (**Figures 6**, **7**). But why there was even no viral protein expression in CSFV-infected 3D4/21^STAT1-/-^ cells? It was important to emphasize that STAT1 plays a crucial role in viral replication; previous studies have shown that knocking down STAT1 significantly inhibits HIV replication (38). CSFV requires a certain concentration of STAT1 for replication, which is not in conflict with the result that STAT1 promotes autophagy. STAT1 overexpression resulted in a reduction of autophagy-associated proteins and autophagy flow, suggesting that STAT1 inhibits autophagy (**Figures 8**, **9**). These findings highlight the complexity of cellular immunity and underscore the potential roles played by STAT1. Notably, autophagy often exhibits dual functions within immunology. On the one hand, it facilitates the elimination of certain pathogens from the body (39); on the other hand, some pathogens evade innate immunity through manipulating the vesicular structure of autophagosomes (40). Additionally, STAT1 has also been implicated in promoting infections related to virus and bacterial diseases (38, 41). Therefore, further exploration into how STAT1 influences autophagy during CSFV infection needs to be conducted given its potential implications for understanding host-pathogen interactions and developing targeted therapeutic strategies. At present, there are many examples of overexpression and overactivation of STAT1 associated with cancer, and various strategies have been employed to develop STAT inhibitors, including interfering with SH2-mediated dimerization, DNA binding, or deactivation and inducing STAT degradation (28). This study showed that the absence or reduction of STAT1 significantly enhanced autophagy during viral infection. An idea to develop new drugs can consider STAT1 as a target, and then select appropriate autophagy regulators to counteract drug resistance. This strategy may enhance treatment efficacy for both viral infections and cancer.

In summary, this study successfully generated PK-15 and 3D4/21 cell lines with a homozygous knockout of the STAT1 gene using a CRISPR/Cas9 editing system mediated by sgRNA. This lays the foundation for future validation of the cell lines’ resistance to CSFV and elucidating the role of STAT1 in autophagy during CSFV infection.

## Data Availability

The original contributions presented in the study are included in the article/supplementary material, Further inquiries can be directed to the corresponding author/s.
